# Latitudinal cogradient variation of development time and growth rate and a negative latitudinal body weight cline in a widely distributed cabbage beetle

**DOI:** 10.1371/journal.pone.0181030

**Published:** 2017-07-12

**Authors:** Jianjun Tang, Haimin He, Chao Chen, Shu Fu, Fangsen Xue

**Affiliations:** 1 College of Computer and Information Engineering, Jiangxi Agricultural University, Nanchang, China; 2 Institute of Entomology, Jiangxi Agricultural University, Nanchang, China; 3 Department of Entomology and Nematology, University of Florida, Gainesville, Florida, United States of America; University of Innsbruck, AUSTRIA

## Abstract

The evolutionary and phenotypic responses to environmental gradients are often assumed to be the same, a phenomenon known as “cogradient variation”. However, only a few insect species display cogradient variation in physiological traits along a latitudinal gradient. We found evidence for such a response in the examination of the life history traits of the cabbage beetle *Colaphellus bowringi* from 6 different geographical populations at 16, 19, 22, 24, 26 and 28°C. Our results showed that larval and pupal development times significantly decreased as rearing temperature increased, and that growth rates were positively correlated with temperature. Body weight tended to decrease with increasing temperature, consistent with the general pattern in ectothermic animals. Larval development time was positively correlated with latitude, whereas the growth rate decreased as latitude increased, showing an example of latitudinal cogradient variation. Body weight significantly decreased with increasing latitude in a stepwise manner, showing a negative latitudinal body weight cline. Females were significantly larger than males, consistent with the female biased sex dimorphism in insects. Body weight tended to decrease with increasing rearing temperature, whereas the differences in sexual size dimorphism (SSD) tended to decrease with increasing body weight, which biased our results toward acceptance of Rensch’s rule. We found that weight loss was an important regulator of SSD, and because male pupae lost significantly more weight at metamorphosis than female pupae, SSD was greater in adults than in pupae. Overall, our data provide a new example that a latitudinal cogradient variation in physiological traits is associated with a negative latitudinal body weight cline.

## Introduction

Studying geographical variation in life history traits of insect species helps us to fully comprehend the evolutionary significance of phenotypic patterns in nature. Comparisons among populations of different geographical origin prove particularly useful because one can assume that there are underlying gradients in the physical environment, including the day length, temperature, and duration of the growing season. Populations along environmental gradients (e.g., latitude, altitude) often show biological gradients in larval development time, body size and growth rate [1–9]. The gradient variations in these traits may be cogradient or countergradient. Cogradient variation (CoGV) describes a geographic pattern of variation in which genetic and environmental influences on phenotypic expression act in the same direction on a trait (termed synergistic selection by Falconer [10]); thus, phenotypic variation is accentuated among populations across the gradient [11]. For example, the common brown butterfly, *Heteronympha merope*, from low latitude (warmer climate) populations has a faster intrinsic growth and development rate than those from higher latitudes (cooler climates) [9]. Countergradient variation (CnGV) describes a geographic pattern of variation in which genetic and environmental influences on phenotypes oppose one another (termed antagonistic selection by Falconer [10]), thereby reducing the phenotypic differentiation between populations [11–13]. For example, larval development times consistently decreased with an increasing latitudinal gradient in four species of geometrid moths, *Cabera exanthemata*, *Cabera pusaria*, *Chiasmia clathrata* and *Lomaspilis marginata* [6]. The lichen-feeding moth, *Eilema depressum*, from a high latitudinal (60° N) population had a higher growth rate than those from a low latitudinal population (46–47° N) [14], and in the generalist grasshopper, *Melanoplus femurrubrum*, northern populations develop more rapidly and show higher growth rates than southern populations [8].

Conover et al. compiled examples of CnGV and CoGV from the literature [11] and found 13 insect species displaying CnGV, mostly in physiological traits (such as developmental time, growth rate and metabolic rate) and occasionally in morphological traits (such as body size). However, only two insect species displayed CoGV in morphological traits (such as wing size) [11]. The fruit fly, *Drosophila melanogaster*, has CnGV variation in a physiological trait coupled with CoGV in a morphological trait [11]. In recent years, 6 species have shown conclusive evidence of CnGV in physiological traits [6,8,14]; thus, CnGV is apparently a more widespread phenomenon than CoGV in insects. An understanding of the evolution of CoGV and CnGV should help predict the consequences of climate change [15]. However, the mechanisms that drive CnGV or CoGV remain unknown.

Clinal variation in body size across a latitudinal gradient may display a Bergmann cline [16], with larger organisms at higher latitudes (a temperature effect), or a converse Bergmann cline [17], with smaller organisms at higher latitudes (a season length effect). A systematic literature review on intraspecific variation in insect size along latitudinal clines was performed. For insects, 123 studies found Bergmann clines, whereas 111 studies found converse Bergmann clines [18]; therefore, Bergmann’s rule cannot be considered a valid ecogeographical law for insects. To date, the cause of Bergmann size clines continues to be disputed; however, converse Bergmann clines could be explained by shorter periods for growth and development at higher latitudes, resulting in decreasing adult size [16,19]. Bergmann and converse Bergmann clines can be interpreted as over- and undercompensating countergradient variation, respectively [11,19]. For examples, the overcompensating CnGV in growth is associated with a Bergmann cline in the ant lion, *Myrmeleon immaculatus* [20]; whereas the undercompensating CnGV in growth is associated with a converse Bergmann cline in the water strider, *Aquarius remigis* [21]. Countergradient selection in development time resulted in Bergmann’s rule in the yellow dung fly, *Scathophaga stercoraria*.

Males and females generally differ in body size, a phenomenon called sexual size dimorphism (SSD). Most insects exhibit sexual size dimorphism, with females larger than males; indeed, only 7% of insect species with SSD have larger males than females [22]. Seventy-two percent of Coleoptera species exhibit female-biased SSD; whereas only 9% of beetle species show male-biased SSD [22]. SSD tends to increase with increasing overall body size when males are the larger sex and decrease with body size when females are the larger sex, a pattern known as Rensch’s rule [23]. However, data from 158 insects show that SSD increases with body size in species with female-biased SSD, a result seemingly inconsistent with Rensch’s rule [24]. More recently, an examination of the temperature–size responses of a wide range of arthropod species (including Diptera, Orthoptera, Lepidoptera, Coleoptera and Calanoida) showed similar body size plasticity to temperature in both the males and females of a species on average [25] without supporting the Rensch’s rule. Differences in body size between males and females among different geographical populations can occur, resulting in latitudinal clines in SSD [4,26–28], which suggest that temperature or other ecological factors that vary with latitude may affect selection on the sexes. Similarly, females and males differences in phenotypic plasticity of size across different temperatures may alter sex size dimorphism [22,28–32]. Therefore, the selective mechanisms driving patterns of SSD remain debatable.

Development time, body size and growth rate are three key associated life history traits. These traits should reflect adaptation to the environment [33]. To date, relatively few studies jointly consider all three variables when studying life history variation along latitudinal gradients [6,8,20,28,34,35]. Here, we collected six geographical populations of the cabbage beetle *Colaphellus bowringi* Baly (Coleoptera: Chrysomelidae) and used their life history patterns to test the relative contributions and phenotypic plasticity levels of response to temperature difference.

## Materials and methods

### Ethics statement

Because the cabbage beetle *C*. *bowringi* is a defoliator pest of crucifers in vegetable gardens in China, no permits were required for collecting the insect and performing the experiments. All experiments were conducted at the Institute of Entomology, Jiangxi Agricultural University, Nanchang, Jiangxi Province (28°46′ N, 115°49′ E).

### Life history of *C*. *bowringi*

The cabbage beetle *C*. *bowringi* is a defoliator pest of crucifers that is widely distributed in China. In LN from Longnan County, XS from Xiushui County, XY from Xinyang County and TA from Taian City populations, the host plants are primarily the cultured radish, *Raphanus sativus* var. *longipinnatus*, and the cultured Chinese cabbage, *Brassica chinensis*. The host plants for SY from Shenyang City and HB from Haerbin City populations are primarily garden cress, *Lepidium apetalum*, and India yellow cress, *Rorippa indica*, which are both generally wild. The life history of *C*. *bowringi* differs among populations. The LN and XS populations have similar annual life histories, showing a short-day response (develops at short day length, but enters diapause at long day length) when mean daily temperature is ≥ 22°C and ≥ 20°C, respectively. All individuals enter diapause when mean daily temperature is ≤ 18°C or ≤ 20°C for LN and XS populations, respectively, both independent of photoperiod [36–38]. In the field, two distinct infestation peaks occur: the spring generation between March and April, and the three autumn generations between September and November, which undergo an aestivating and hibernating imaginal diapause in the soil [36–38]. The XY and TA populations experience similar annual life histories with two distinct infestation peaks: the spring generation between April and early May, and the autumn generation between late August and mid-October, which undergoes an imaginal summer and winter diapause in the soil. The incidence of diapause in the two populations is determined by temperature and is independent of photoperiod. All individuals enter diapause at temperatures below 25°C [38–39]. The SY and HB populations have an annual life history; one generation is produced in summer between May and June that will overwinter as diapausing adults in the soil. Diapause incidence is determined by temperature and is independent of photoperiod; almost all individuals enter diapause at 28°C [38].

### Insect culture

**Fig 1:**
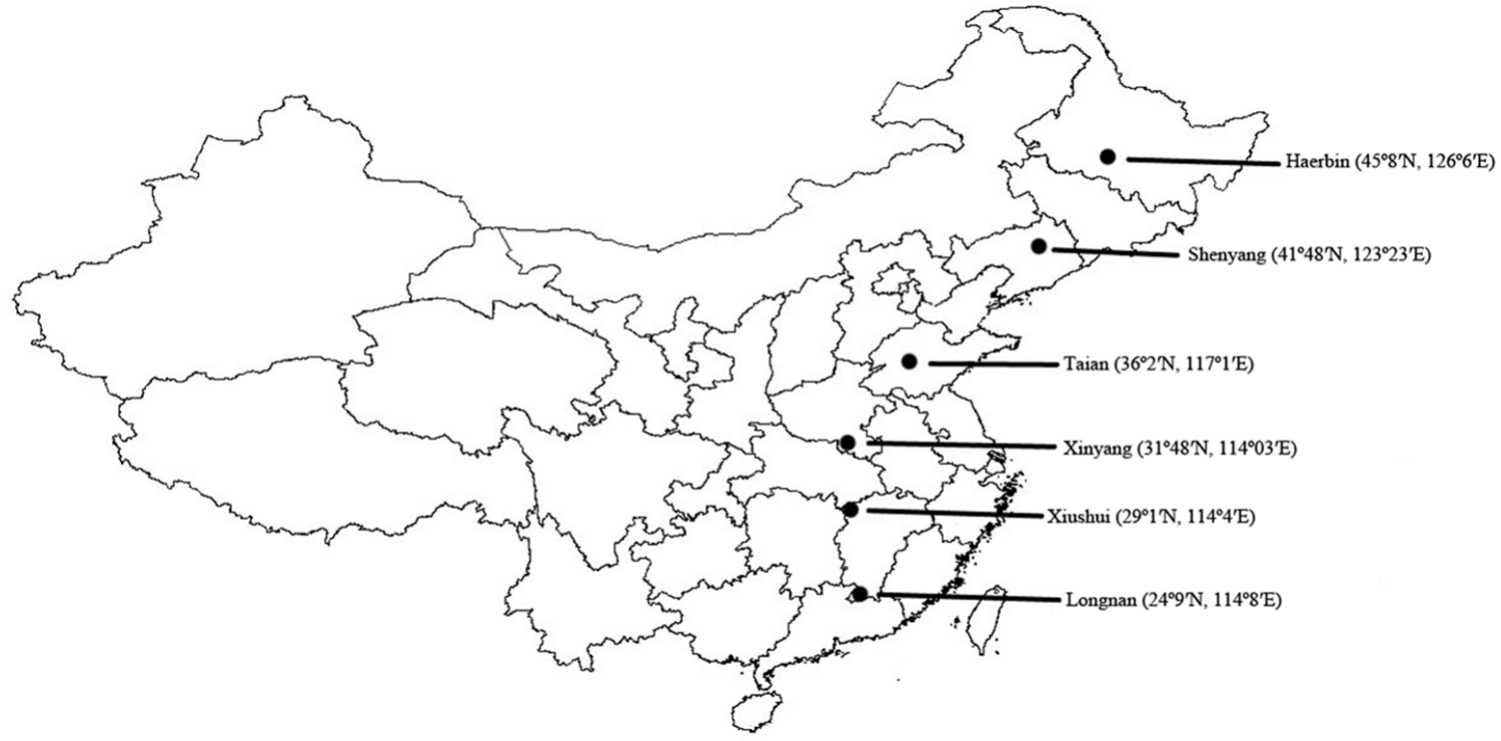
Locations of sample collection sites of *Colaphellus bowringi*.

Naturally diapausing adults of the six *C*. *bowringi* populations from Longnan County (LN, 24°9' N, 114°8' E), Xiushui County (XS, 29°1' N, 114°4' E), Xinyang County (XY, 31°48' N, 114°03' E), Taian City (TA, 36°2' N, 117°1' E), Shenyang City (SY, 41°48' N, 123°23' E) and Haerbin City (HB, 45°8' N, 126°6' E) were collected from vegetable gardens between late March and June 2014 (Fig 1). These diapausing adults were transferred to large glass bottles (50 cm diameter, 180 cm height) containing soil in which they could burrow and become dormancy. Those bottles were placed outdoors at Jiangxi Agricultural University, Nanchang, Jiangxi Province (28°46' N, 115°59' E). The post-diapause adults for the six populations used for this study emerged from the soil between late February and early March 2015. Female and male for each population were paired randomly in 30 to 40 petri dishes (9.0 cm diameter, 2.0 cm height) lined with filter paper and fresh Chinese cabbage leaves of *B*. *chinensis* for mating and oviposition. Eggs laid on the first 3 days were collected and newly hatched larvae were transferred to rearing boxes (16 × 11 × 5.5 cm) with fresh Chinese cabbage leaves as experimental materials. Each box contained at least 30 individuals. Three to six replicates for each temperature and population were observed daily and supplied with new food as required. All experiments were conducted in illuminated incubators (LRH-250-GS; Guangdong Medical Appliances Plant, Guangdong, China). The light intensity during photophase was approximately 1.97 W m^**-2**^, and the variation in temperature was ± 1°C.

### Experimental arrangement

Newly hatched larvae were reared at six temperatures (16, 19, 22, 24, 26 and 28°C) under a long photoperiod (L16:D8 photocycle). When larvae matured, they were placed individually in cell culture plates with 12 holes (holes: diameter, 2.4 cm; height, 2 cm) for pupation and eclosion. At least 8 cell culture plates were used for each temperature and population. For each individual, we measured the following traits: development time from hatching to pupation and to adult eclosion, pupal and adult weights, growth rate, proportional weight loss at metamorphosis, and SSD. Pupae were weighed on the day following pupation; whereas adults were weighed on the day of eclosion, after hardening of the elytra. We used an electric balance (AUY120, SHIMADZU Corporation, Japan).

Growth rate was calculated as ln pupal weight / larval development time [3]. Weight loss between pupation and adult eclosion was calculated using the formula: Proportion weight lost = l−(adult weight / pupal weight). SSD for pupae was estimated for each population and temperature using the Lovich and Gibbons [40] index in which SSD = (size of the larger sex/size of the smaller sex)−1. Overall, 4591 offspring from six populations were raised to adulthood and weighed.

### Statistical analyses

Statistical analyses were conducted using the SPSS 17.0 statistical software package (IBM, www.ibm.com). For our analysis of life history traits we used mixed linear model, in which temperature, population and sex were treated as fixed main effects and rearing box was treated as a random effect. The full model with all possible interaction terms was performed at first, the non-significant three-way term (temperature-by-population-by-sex) was dropped from the final model in the analysis.

One-way ANOVA was used to determine whether there were significant differences in life history traits in different populations at each temperature. One-way analysis of variance (ANOVA) and Tukey’s tests were used to compare the differences in life history traits between sexes in each population and at each temperature. Throughout the text, all means are ± 1 SE.

## Results

### Development time in relation to temperature and population

**Fig 2:**
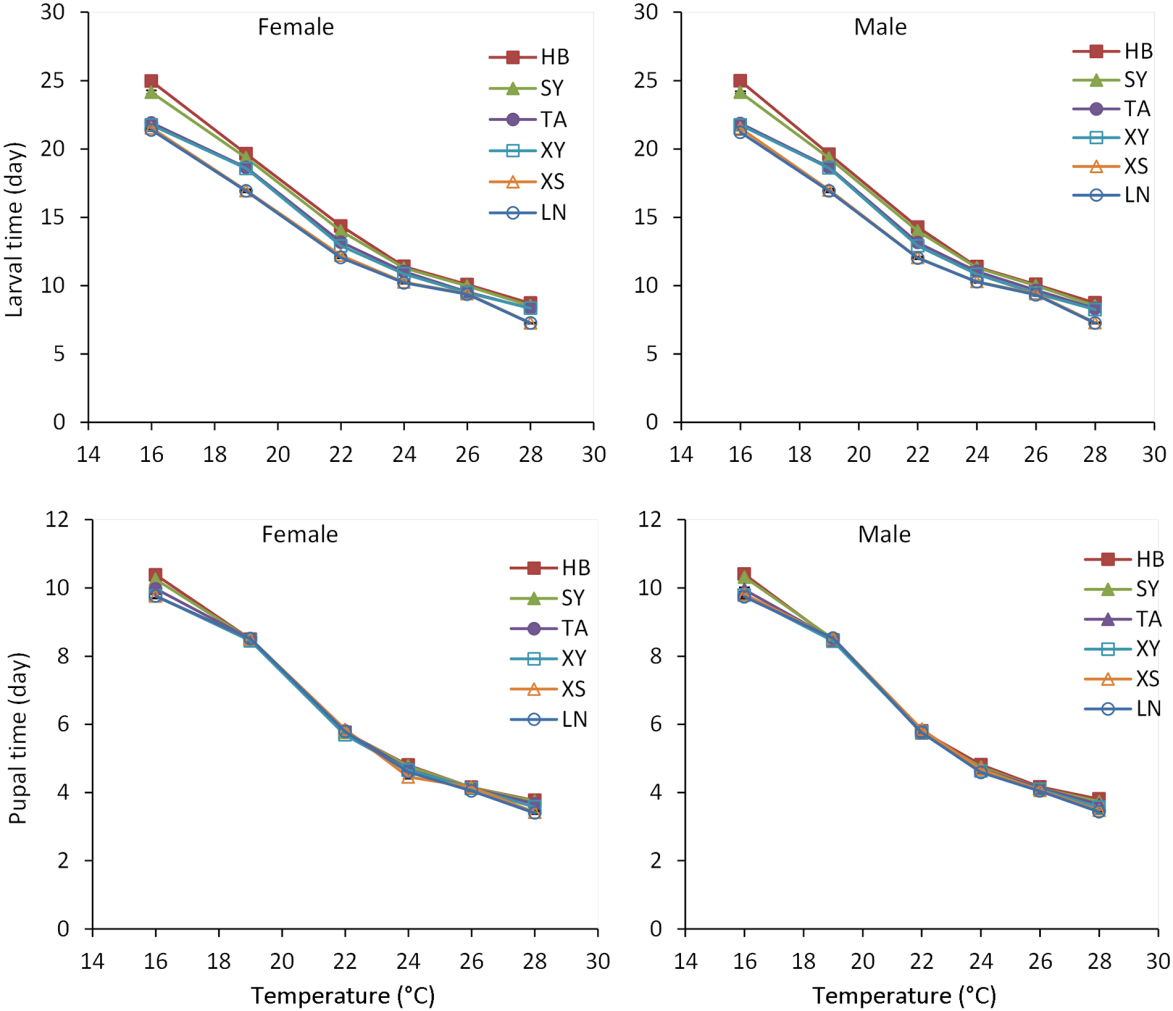
Larval and pupal development times of *Colaphellus bowringi* females and males in relation to temperature and population (Populations: LN, Longnan; XS, Xiushui; XY, Xinyang; TA, Taian; SY, Shenyang; HB, Haerbin). Error bars indicate SE.

Temperature and population significantly affected development time (Table 1). In all populations and for both sexes, larval and pupal development times significantly decreased as rearing temperature increased (Fig 2; Table 1, for temperature main effect; see also S1 Table). At all temperatures, larval development time gradually increased as latitude of origin increased (Fig 2; Table 1, for population main effect; see also S2 Table), which is an example of latitudinal cogradient. However, significant differences in pupal development time among populations were only found at 16 and 24°C and did not exhibit a latitudinal gradient (S1 Table).

**Table 1:** Results from linear mixed model analyses of fixed effects on larval time, pupal time, pupal weight and growth rate in *Colaphellus bowringi* in relation to temperature, population and sex.

Traits	Fixed effects	Num. d.f	Den. d.f	F	*P*
Larval time	Temperature	5	113.012	3742.143	< 0.001
	Population	5	112.979	85.091	< 0.001
	Sex	1	4435.609	1.934	.164
	Population × Temperature	25	112.979	5.623	< 0.001
	Temperature × Sex	5	4435.253	.618	.686
	Population × Sex	5	4435.568	.284	.922
Pupal time	Temperature	5	112.773	3099.851	< 0.001
	Population	5	112.614	3.985	.002
	Sex	1	4454.389	.112	.738
	Population × Temperature	25	112.597	1.034	.432
	Temperature × Sex	5	4452.599	1.373	.231
	Population × Sex	5	4454.145	1.295	.263
Pupal weight	Temperature	5	118.127	55.702	< 0.001
	Population	5	117.828	1156.393	< 0.001
	Sex	1	4482.209	9003.503	< 0.001
	Population × Temperature	25	117.698	8.339	< 0.001
	Temperature × Sex	5	4478.818	2.104	.062
	Population × Sex	5	4481.514	56.768	< 0.001
Growth rate	Temperature	5	115.210	2991.125	< 0.001
	Population	5	115.140	327.062	< 0.001
	Sex	1	4440.326	3446.953	< 0.001
	Population × Temperature	25	115.139	8.357	< 0.001
	Temperature × Sex	5	4439.581	93.541	< 0.001
	Population × Sex	5	4440.237	4.744	< 0.001

### Growth rate in relation to temperature, population and sex

Temperature, population, sex, and their interactions (temperature × population, temperature × sex, population × sex) significantly affected larval growth rate (Table 1). Growth rate increased significantly as rearing temperature increased, regardless of population or sex (Fig 2; Table 1, for temperature main effect; see also S1 Table). Growth rate significantly decreased as latitude increased, suggesting a latitudinal cogradient (Fig 2; Table 1, for population main effect; see also S2 Table). Female growth rate was significantly higher than male growth rate in all populations and at all temperatures (S3 Table, *P* < 0.05).

### Body weight and weight loss in relation to temperature, population and sex

**Fig 3:**
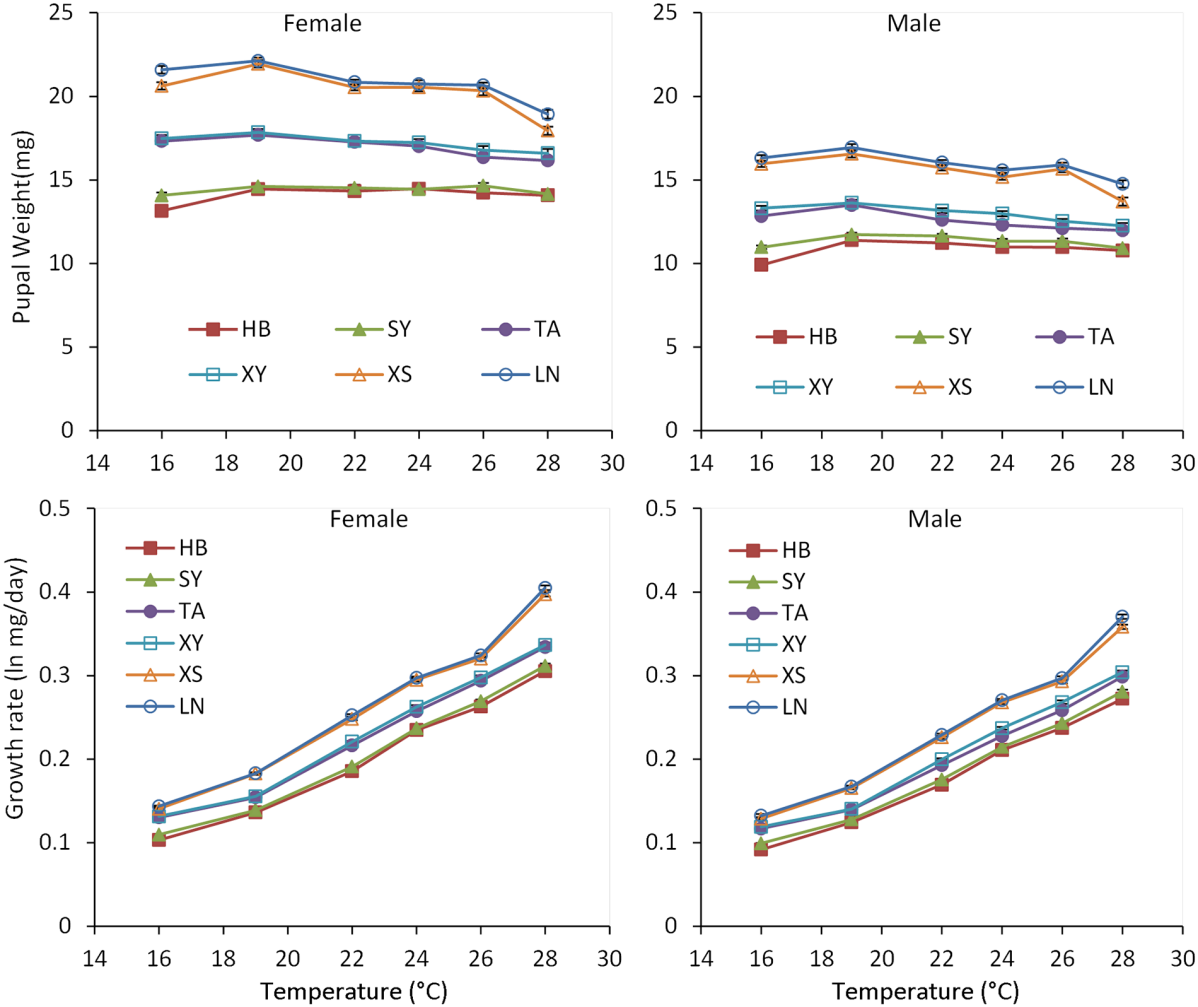
Pupal weight and larval growth rate of *Colaphellus bowringi* females and males in relation to temperature and population (Populations: LN, Longnan; XS, Xiushui; XY, Xinyang; TA, Taian; SY, Shenyang; HB, Haerbin). Error bars indicate SE.

**Fig 4:**
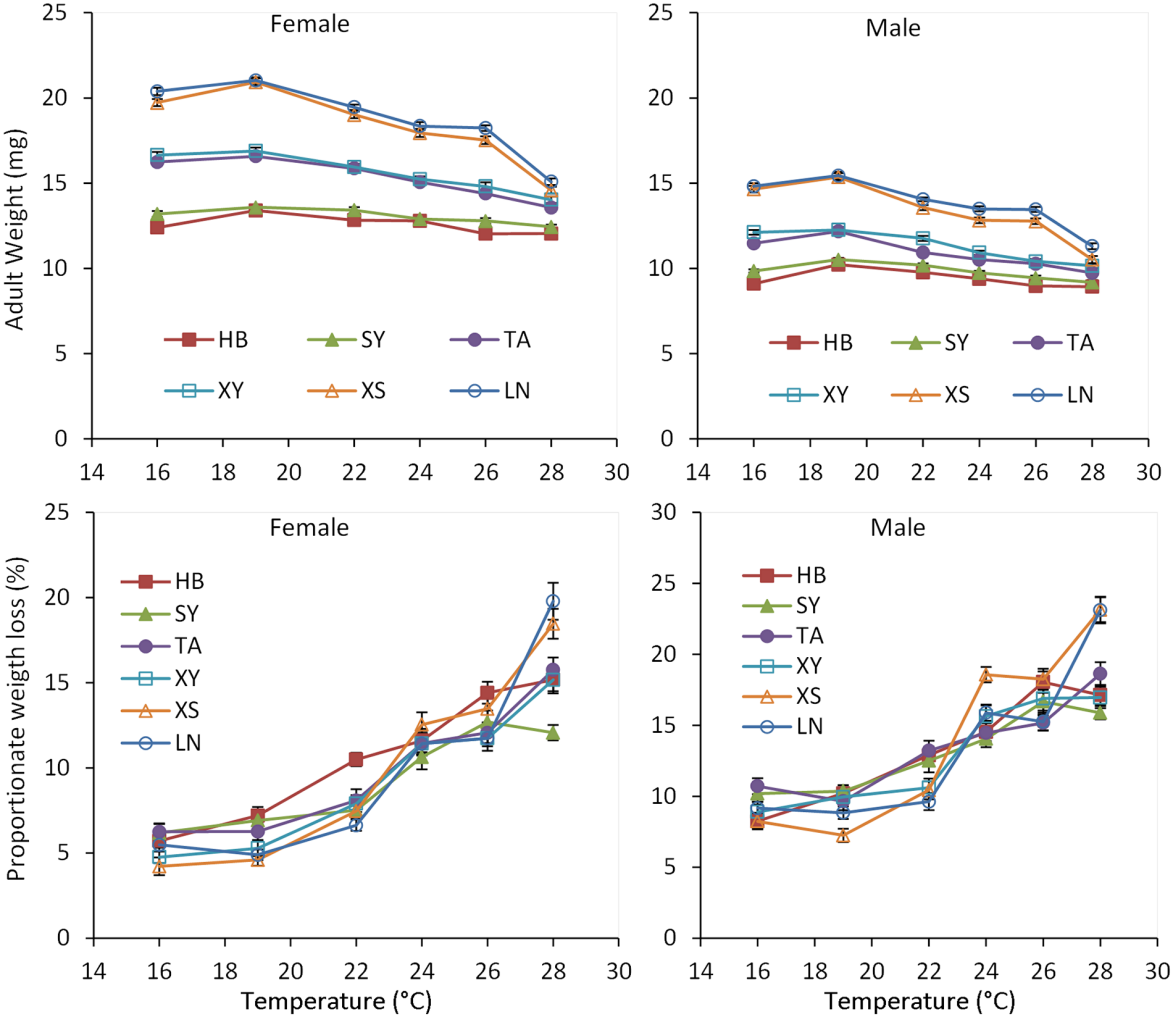
Adult weight and proportionate weight loss of *Colaphellus bowringi* females and males in relation to temperature and population (Populations: LN, Longnan; XS, Xiushui; XY, Xinyang; TA, Taian; SY: Shenyang; HB, Haerbin). Error bars indicate SE.

**Fig 5:**
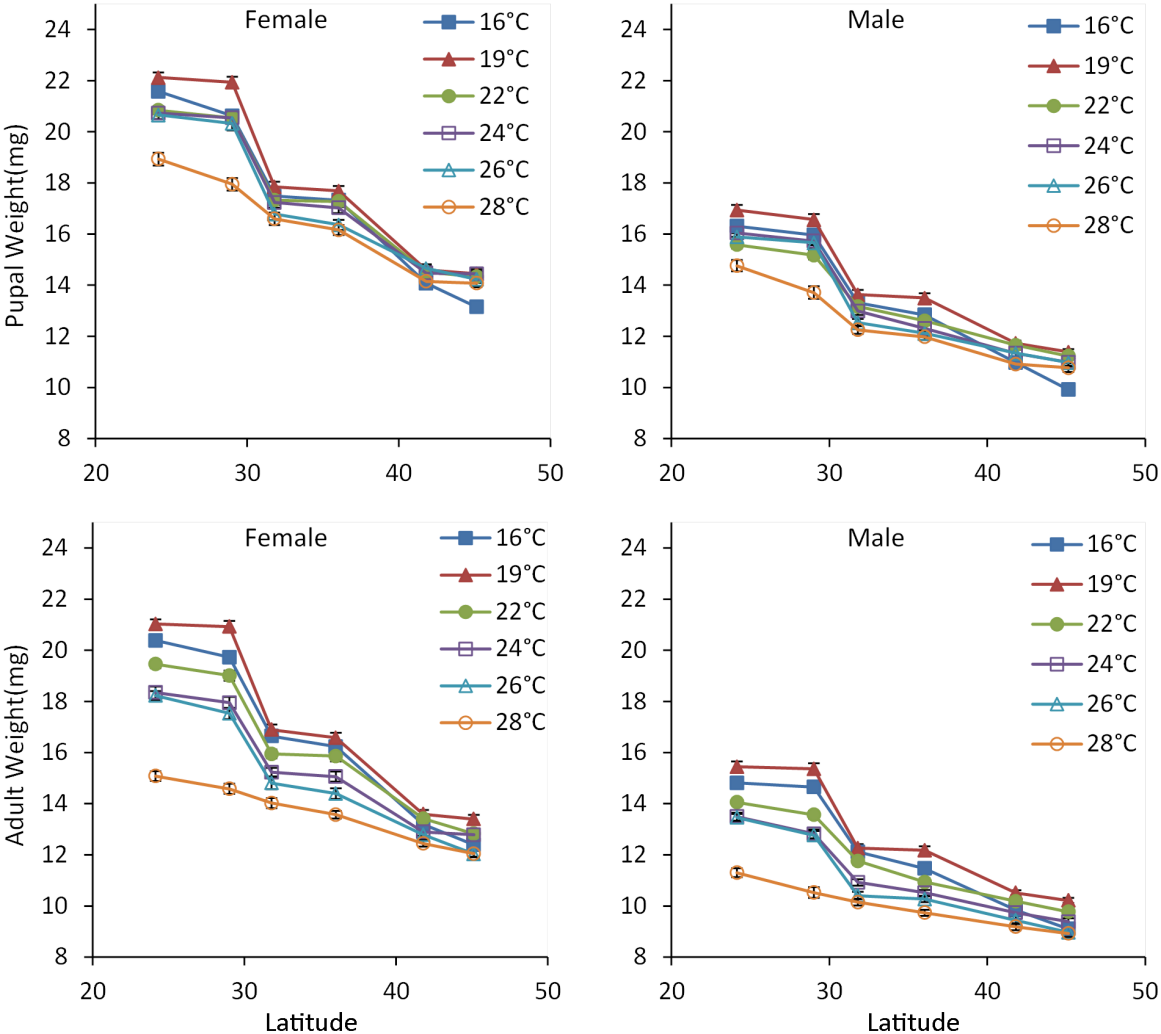
Latitudinal variations of body weights in *Colaphellus bowringi* at different temperatures (24°9' N, 114°8' E for LN; 29°1' N, 114°4' E for XS; 31°48' N, 114°03' E for XY; 36°2' N, 117°1' E for TA; 41°48' N, 123°23' E for SY; 45°8' N, 126°6' E for HB). Error bars indicate SE.

Body weights for both pupae and adults were significantly affected by temperature, population, sex, and their interactions (temperature × population, temperature × sex, population × sex; Tables 1 and 2). Pupal and adult weights in all populations gradually decreased as rearing temperature increased from 19 to 28°C, consistent with the general pattern in ectothermic animals [41] (Figs 3 and 4; Tables 1 and 2, for temperature main effect). However, the response of body weight to temperature was different among populations. For example, the pupal body weight of females from the northern HB and SY populations did not significantly differ at any temperature between 19 and 28°C (see S1 Table, *P* > 0.05), whereas pupal body weight in the southern LN and XS populations differed significantly across the same temperature range (see S1 Table, *P* < 0.05). The body weights for both pupae and adults significantly decreased as latitude increased (Tables 1 and 2, for population main effect), showing a stepwise descent cline (Fig 5). Females were significantly larger than males (see also S3 Table, *P* < 0.05). Comparisons of body weight for all populations at each temperature revealed that female pupae were from 24.5% to 38.2% heavier than male pupae, whereas female adults were from 29.3% to 45.1% heavier than male adults.

**Table 2:** Results from linear mixed model analyses of fixed effects on adult weight and proportionate weight loss in *Colaphellus bowringi* in relation to temperature, population and sex.

Traits	Fixed effects	Num. d.f	Den. d.f	F	*P*
Adult weight	Temperature	5	114.113	275.823	< 0.001
	Population	5	113.807	1118.377	< 0.001
	Sex	1	4484.199	10637.688	< 0.001
	Population × Temperature	25	113.644	20.458	< 0.001
	Temperature × Sex	5	4480.406	12.385	< 0.001
	Population × Sex	5	4483.349	70.824	< 0.001
Proportionate weight loss	Temperature	5	114.087	187.165	< 0.001
	Population	5	113.874	1.935	.094
	Sex	1	4464.532	629.639	< 0.001
	Population × Temperature	25	113.832	5.397	< 0.001
	Temperature × Sex	5	4462.103	.836	.524
	Population × Sex	5	4464.156	2.065	.067

Temperature, sex, and the interaction of temperature and population significantly affected weight loss from pupa to adult (Table 2). Weight loss increased significantly as rearing temperature increased (Fig 5). Males from the southern LN and XS populations lost significantly more weight at 28°C (23.1% and 23.2%, respectively) than males from northern HB and SY populations (15.2% and 17.1%, respectively) (see S2 Table, *P* < 0.05). Male pupae lost significantly more weight at metamorphosis than those of females in almost all treatments (see S3 Table, *P* < 0.05).

### Sexual size dimorphism

**Fig 6:**
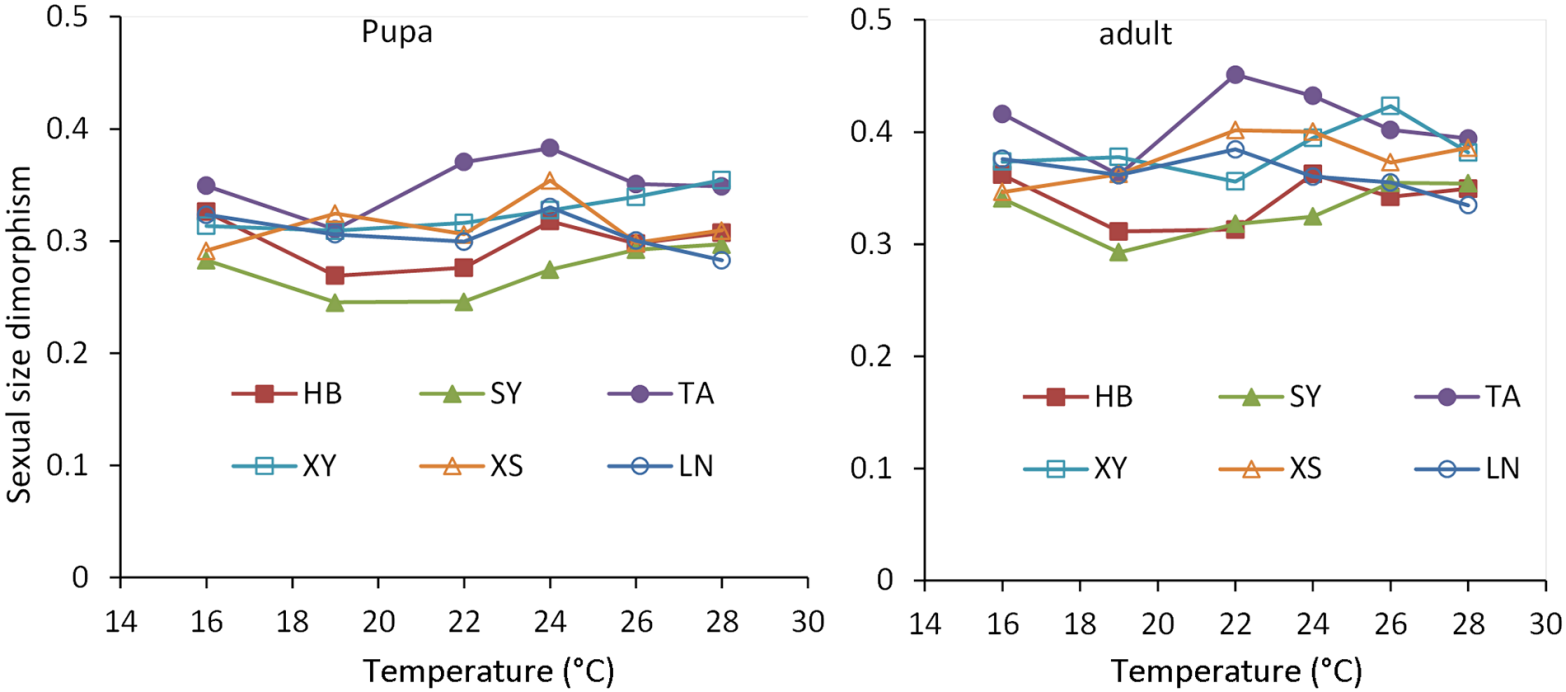
Sexual size dimorphism of populations of *Colaphellus bowringi* reared at six different temperatures (16, 19, 22, 24, 26 and 28°C). Sexual dimorphism was estimated for each population at each temperature as (mean size of the larger sex (mg)/mean size of the smaller sex (mg) − 1).

The magnitude of SSD for pupae and adults is shown in Fig 6. Among temperatures, SSD was lowest at 19°C in HB (0.26 for pupae, 0.31 for adults), SY (0.25 for pupae, 0.29 for adults), TA (0.31 for pupae, 0.36 for adults) and XY (0.31 for adults) populations. The XS population showed the lowest SSD at 16°C (0.29 for pupae, 0.34 for adults). The LN population showed the lowest SSD at 28°C (0.28 for pupae, 0.31 for adults). SSD for adults was greater at relatively high temperatures, although size dimorphism for all populations did not show a gradient change with temperature. For example, the highest SSD occurred at 22°C for LN and TA populations, at 24°C for XS and HB populations, and at 26°C for XY and SY populations. SSD of pupae and adults from the southern LN and XS populations was higher than the SSD from the northern SY and HB populations at both 19 and 22°C. SSD in the adult stage was greater than that at the beginning of the pupal phase due to male pupae losing significantly more weight at metamorphosis than female pupae (Figs 5 and 6).

## Discussion

Although ecologists often postulate that the evolutionary response to an environmental gradient will be the same as the phenotypic response (cogradient variation), few studies with insects have provided evidence of such an evolutionary response [9,11]. In most studies, the evolutionary response to a gradient is the opposite of the ecological response (countergradient variation) [6,8,11,14]. In the present study, *C*. *bowringi* provided a strong example of CoGV in physiological traits along a latitudinal gradient (Figs 2 and 3). Larval development time gradually increased with increasing latitude and growth rate was negatively correlated with latitude, which suggested that individuals from southern populations had intrinsically faster growth and development than those from northern populations. This result suggested a positive covariation between genotypes and environments across a latitudinal gradient. Variation in larval growth and development may be associated with gradual changes in seasonality across the landscape [17,42–43]. However, whether gradual changes in seasonality along the latitudinal gradient were the driving factor of cogradient variation in *C*. *bowringi* is uncertain. The southern and northern populations experience completely different seasonality at the larval stage. The southern LN and XS populations and the intermediate latitude XY and TA populations undergo summer diapause, and therefore, the larvae in these four populations grow and develop primarily in spring and autumn, experiencing gradually increasing spring temperatures and gradually decreasing autumn temperatures. However, the larvae of northern HB and SY populations experience gradually increasing summer temperatures between late May and June. Given the differences in seasonality between the populations, the latitudinal CoGV in development time and growth rate in *C*. *bowringi* should not be associated with gradual changes in seasonality. The CoGV may be genetically based, caused by selection imposed by local climatic conditions experienced by larvae.

Given that larval development time of *C*. *bowringi* gradually increased with increasing latitude and that body weights gradually decreased with increasing rearing temperature from 19 to 28°C, we would expect to find a positive relationship between latitude and body weight along the latitudinal gradient because generally, a positive relationship is found between development time and body size in ectotherms [44]. In the present study, we did not measure the adult body size of *C*. *bowringi* collected from fields, but we measured body weight for both females and males of their offspring and analyzed latitudinal variation in body weight. We found that beetle displayed a negative latitudinal body weight cline, that is, body weight decreased in a stepwise manner with increasing latitude (Fig 5). A converse Bergmann’s size cline may be more likely in organisms whose life cycles are linked to length of the growing season [2,17,19,45–47]. Because season lengths tend to be longer at lower latitudes, organisms that display a converse Bergmann cline may have more time to grow to large sizes. However, the negative latitudinal body weight cline occurred in *C*. *bowringi* showed that the southern populations had significantly shorter larval development time and significantly heavier body weight than the northern populations. Therefore, the primary factor driving the pattern of the negative latitudinal body weight cline in *C*. *bowringi* was not the season length. Southern and northern populations experience completely different seasonal climates, with the southern populations experiencing spring and autumn climates with a summer diapause, whereas northern populations experience a summer climate. The negative latitudinal body weight cline in *C*. *bowringi* was most likely associated with local climate conditions. Additionally, the southern populations primarily feed on the cultured crucifers, whereas the northern HB and SY populations primarily feed on the wild crucifers (garden cress and India yellow cress), which might contribute to the negative latitudinal body weight cline [48]. Recent study from published data compilations showed that voltinism was significantly related to body mass in terrestrial species, with larger species often having a single generation and smaller species producing multiple generations annually [49]. In contrast to this result, our study revealed that the univoltine northern HB and SY populations had smaller body weight, whereas the multivoltine southern LN and XS populations showed greater body weight.

The southern LN and XS populations exhibited a shorter larval development time and greater body weight than the northern HB and SY populations. This difference suggested that the genetically determined body weight of southern and northern populations might have evolved independently. Our data provide a new example that the negative latitudinal body weight cline is associated with CoGV, with shorter development times at lower latitudes.

Only two species have displayed a stepwise Bergmann cline along a latitudinal gradient [18]. The negative latitudinal body weight cline displayed in *C*. *bowringi* had a distinct downward stepwise transformation at temperatures between 16 and 26°C (Fig 5). The low latitude LN and XS populations have a similar annual life history (summer diapause and photoperiodic response) and showed the greatest body weight. The intermediate latitude XY and TA populations have a similar annual life history but lacked a photoperiodic response and displayed intermediate body weights. The high latitudinal SY and HB populations did not have a summer diapause or photoperiodic response and exhibited the lowest body weights. Such a stepwise transformation might reflect different evolutionary origins of the responses to local environmental conditions in the different geographical populations.

Consistent with female-biased dimorphism in insects, the pupae and adults of female *C*. *bowringi* were significantly larger than male pupae and adults. According to Rensch’s rule, when females are the larger sex, SSD should decrease as body size increases [29]. In this study, as rearing temperature increased, the body weight of *C*. *bowringi* tended to decrease, whereas the differences in SSD tended to decrease with increasing body weight. With this result, we were biased toward acceptance of Rensch’s rule. Similar results are also reported in a seed-feeding beetle, *Callosobruchus maculates*, and a butterfly *Lycaena tityrus* [45,50]. Regardless of temperature, SSD varied among populations, indicating differences in the genetically determined body size of males and females of different populations. The SSD for all populations changed with temperature, likely due to a sex difference in plasticity of body size (Fig 6). When we compared the difference in SSD between pupae and the adults, SSD was lower in pupae than in adults for all populations at each temperature because the male pupae lost significantly more weight at metamorphosis than the female pupae (Table 2; *P* < 0.05), which suggested that weight loss was an important regulator of SSD [51].

## Supporting information

S1 Table A comparison of life-history traits (mean ± 1 SE) for females and males among temperatures. (DOC)

S2 Table A comparison life-history traits (mean ± 1 SE) for females and males among populations. (DOC)

S3 Table A comparison of life-history traits (mean ± 1 SE) between sexes. (DOC)
