# Supplementary material for: Latitudinal cogradient variation of development time and growth rate and a negative latitudinal body weight cline in a widely distributed cabbage beetle
Source: PLoS One. 2017 Jul 12;12(7):e0181030. doi: 10.1371/journal.pone.0181030 (PMC5507546; doi:10.1371/journal.pone.0181030)
Supplement: S2 Table — (DOC) [file pone.0181030.s002.doc]

**S2 Table. A comparison life-history traits (mean ± 1 SE) for females and males among populations. Figures followed by the same letter do not differ significantly among populations at the each temperature (One-way analysis of variance (ANOVA) and Tukey’s test, threshold for significance *P* < 0.05)**

| Temperature and trait | HB | |  | | SY | |  | | TA | |  | | XY | |  | | XS | |  | | LN | |
| --- | --- | --- | --- | --- | --- | --- | --- | --- | --- | --- | --- | --- | --- | --- | --- | --- | --- | --- | --- | --- | --- | --- |
|  | Female | Male | | Female | | Male | | Female | | Male | | Female | | Male | | Female | | Male | | Female | | Male |
| 16 °C | N=42 | N=47 | | N=55 | | N=57 | | N=40 | | N=42 | | N=47 | | N=51 | | N=109 | | N=108 | | N=71 | | N=73 |
| Larval time (d) | 24.95±0.1a | 24.98±0.09a | | 24.15±0.14b | | 24.14±0.09b | | 21.9±0.11c | | 21.86±0.11cd | | 21.74±0.09cd | | 21.75±0.11cd | | 21.52±0.08cde | | 21.52±0.08cde | | 21.38±0.08de | | 21.22±0.17e |
| Pupal time (d) | 10.38±0.06a | 10.4±0.06a | | 10.25±0.07ab | | 10.32±0.07a | | 9.98±0.07bc | | 9.95±0.06bc | | 9.77±0.06c | | 9.78±0.05c | | 9.76±0.06c | | 9.81±0.07c | | 9.76±0.07c | | 9.74±0.06c |
|  |  |  | |  | |  | |  | |  | |  | |  | |  | |  | |  | |  |
| 19 °C | N=79 | N=85 | | N=74 | | N=75 | | N=71 | | N=79 | | N=76 | | N=80 | | N=87 | | N=88 | | N=75 | | N=78 |
| Larval time (d) | 19.65±0.15a | 19.61±0.14a | | 19.35±0.15a | | 19.33±0.16a | | 18.65±0.11b | | 18.66±0.12b | | 18.57±0.13b | | 18.6±0.08b | | 16.94±0.11c | | 16.99±0.11c | | 16.92±0.07c | | 16.91±0.08c |
| Pupal time (d) | 8.49±0.07a | 8.47±0.06a | | 8.49±0.06a | | 8.51±0.06a | | 8.51±0.06a | | 8.49±0.06a | | 8.45±0.06a | | 8.44±0.06a | | 8.51±0.06a | | 8.52±0.06a | | 8.51±0.06a | | 8.53±0.06a |
|  |  |  | |  | |  | |  | |  | |  | |  | |  | |  | |  | |  |
| 22 °C | N=50 | N=46 | | N=51 | | N=50 | | N=56 | | N=53 | | N=45 | | N=52 | | N=93 | | N=96 | | N=56 | | N=59 |
| Larval time (d) | 14.36±0.07a | 14.28±0.08ab | | 14±0.06b | | 14±0.07b | | 13.18±0.08c | | 13.15±0.08c | | 12.91±0.05c | | 12.9±0.04c | | 12.2±0.07d | | 12.05±0.08d | | 12.04±0.06d | | 12.01±0.07d |
| Pupal time (d) | 5.76±0.08a | 5.8±0.08a | | 5.76±0.07a | | 5.74±0.07a | | 5.8±0.07a | | 5.75±0.07a | | 5.69±0.08a | | 5.77±0.07a | | 5.85±0.06a | | 5.85±0.05a | | 5.79±0.06a | | 5.76±0.06a |
|  |  |  | |  | |  | |  | |  | |  | |  | |  | |  | |  | |  |
| 24 °C | N=58 | N=62 | | N=60 | | N=61 | | N=60 | | N=56 | | N=51 | | N=53 | | N=67 | | N=71 | | N=54 | | N=56 |
| Larval time (d) | 11.4±0.06a | 11.37±0.06a | | 11.28±0.06abc | | 11.31±0.06ac | | 11.03±0.07bd | | 11.04±0.07bcd | | 10.85±0.05d | | 10.82±0.05d | | 10.25±0.06e | | 10.3±0.06e | | 10.2±0.05e | | 10.27±0.06e |
| Pupal time (d) | 4.8±0.04ac | 4.81±0.03a | | 4.78±0.04ac | | 4.75±0.04ac | | 4.68±0.05ac | | 4.69±0.05ac | | 4.66±0.05abc | | 4.63±0.05abc | | 4.46±0.05b | | 4.68±0.04ac | | 4.6±0.04abc | | 4.59±0.04bc |
|  |  |  | |  | |  | |  | |  | |  | |  | |  | |  | |  | |  |
| 26 °C | N=40 | N=46 | | N=40 | | N=40 | | N=63 | | N=62 | | N=58 | | N=55 | | N=97 | | N=103 | | N=52 | | N=58 |
| Larval time (d) | 10.08±0.07a | 10.09±0.06a | | 9.98±0.05a | | 9.99±0.05a | | 9.53±0.07bc | | 9.66±0.06b | | 9.48±0.06bc | | 9.44±0.06bc | | 9.41±0.06bc | | 9.4±0.06bc | | 9.37±0.06bc | | 9.33±0.06c |
| Pupal time (d) | 4.15±0.05a | 4.16±0.05a | | 4.14±0.04a | | 4.13±0.04a | | 4.13±0.04a | | 4.12±0.04a | | 4.12±0.04a | | 4.09±0.04a | | 4.15±0.03a | | 4.06±0.02a | | 4.05±0.05a | | 4.04±0.03a |
|  |  |  | |  | |  | |  | |  | |  | |  | |  | |  | |  | |  |
| 28 °C | N=62 | N=81 | | N=40 | | N=44 | | N=53 | | N=55 | | N=40 | | N=45 | | N=95 | | N=101 | | N=76 | | N=80 |
| Larval time (d) | 8.71±0.06a | 8.73±0.06a | | 8.53±0.06ab | | 8.52±0.06ab | | 8.34±0.06bc | | 8.33±0.06bc | | 8.35±0.06bc | | 8.24±0.06c | | 7.26±0.03d | | 7.29±0.03d | | 7.26±0.03d | | 7.26±0.03d |
| Pupal time (d) | 3.77±0.03a | 3.81±0.04a | | 3.75±0.03a | | 3.74±0.04a | | 3.67±0.06a | | 3.65±0.05a | | 3.58±0.05a | | 3.57±0.05a | | 3.42±0.05a | | 3.48±0.05a | | 3.4±0.03a | | 3.43±0.05a |

Continued

| Temperature and trait | HB | |  | | SY | |  | | TA | |  | | XY | |  | | XS | |  | | LN | |
| --- | --- | --- | --- | --- | --- | --- | --- | --- | --- | --- | --- | --- | --- | --- | --- | --- | --- | --- | --- | --- | --- | --- |
|  | Female | Male | | Female | | Male | | Female | | Male | | Female | | Male | | Female | | Male | | Female | | Male |
| 16 °C | N=42 | N=47 | | N=55 | | N=57 | | N=40 | | N=42 | | N=47 | | N=51 | | N=109 | | N=108 | | N=71 | | N=73 |
| Pupal weight (mg) | 13.15±0.14a | 9.91±0.11b | | 14.07±0.18c | | 10.97±0.1d | | 17.32±0.2e | | 12.84±0.14a | | 17.48±0.21e | | 13.31±0.14ac | | 20.62±0.22f | | 15.96±0.19g | | 21.58±0.23h | | 16.31±0.19g |
| Growth rate(In mg/d) | 0.1±0.001a | 0.09±0.001b | | 0.11±0.001c | | 0.1±0a | | 0.13±0.001d | | 0.12±0.001e | | 0.13±0.001d | | 0.12±0.001e | | 0.14±0.001f | | 0.13±0.001d | | 0.14±0.001f | | 0.13±0.002d |
|  |  |  | |  | |  | |  | |  | |  | |  | |  | |  | |  | |  |
| 19 °C | N=79 | N=85 | | N=74 | | N=75 | | N=71 | | N=79 | | N=76 | | N=80 | | N=87 | | N=88 | | N=75 | | N=78 |
| Pupal weight (mg) | 14.45±0.18a | 11.39±0.11b | | 14.6±0.17a | | 11.73±0.1b | | 17.69±0.2cd | | 13.5±0.18e | | 17.85±0.21c | | 13.63±0.18e | | 21.94±0.21f | | 16.56±0.22g | | 22.12±0.2f | | 16.94±0.2dg |
| Growth rate(In mg/d) | 0.14±0.001a | 0.12±0.001b | | 0.14±0.001a | | 0.13±0.001b | | 0.15±0.001c | | 0.14±0.001a | | 0.16±0.001c | | 0.14±0.001a | | 0.18±0.001d | | 0.17±0.001e | | 0.18±0.001d | | 0.17±0.001e |
|  |  |  | |  | |  | |  | |  | |  | |  | |  | |  | |  | |  |
| 22 °C | N=50 | N=46 | | N=51 | | N=50 | | N=56 | | N=53 | | N=45 | | N=52 | | N=93 | | N=96 | | N=56 | | N=59 |
| Pupal weight (mg) | 14.33±0.18a | 11.23±0.12b | | 14.52±0.19ac | | 11.65±0.11b | | 17.28±0.22d | | 12.61±0.15e | | 17.32±0.18d | | 13.16±0.14e | | 20.54±0.18f | | 15.17±0.17cg | | 20.84±0.16f | | 15.58±0.15g |
| Growth rate(In mg/d) | 0.19±0.001a | 0.17±0.001b | | 0.19±0.001ac | | 0.18±0.001b | | 0.22±0.002d | | 0.19±0.001c | | 0.22±0.001de | | 0.2±0.001f | | 0.25±0.001g | | 0.23±0.002eh | | 0.25±0.001g | | 0.23±0.002h |
|  |  |  | |  | |  | |  | |  | |  | |  | |  | |  | |  | |  |
| 24 °C | N=58 | N=62 | | N=60 | | N=61 | | N=60 | | N=56 | | N=51 | | N=53 | | N=67 | | N=71 | | N=54 | | N=56 |
| Pupal weight (mg) | 14.47±0.11a | 10.98±0.14b | | 14.45±0.14a | | 11.33±0.14b | | 17.02±0.2c | | 12.31±0.12d | | 17.24±0.21c | | 12.98±0.15d | | 20.54±0.23e | | 15.72±0.16f | | 20.73±0.24e | | 16.04±0.15f |
| Growth rate(In mg/d) | 0.23±0.002ab | 0.21±0.002c | | 0.24±0.001a | | 0.21±0.001c | | 0.26±0.002d | | 0.23±0.002b | | 0.26±0.002de | | 0.24±0.002a | | 0.29±0.002f | | 0.27±0.002e | | 0.3±0.002f | | 0.27±0.002e |
|  |  |  | |  | |  | |  | |  | |  | |  | |  | |  | |  | |  |
| 26 °C | N=40 | N=46 | | N=40 | | N=40 | | N=63 | | N=62 | | N=58 | | N=55 | | N=97 | | N=103 | | N=52 | | N=58 |
| Pupal weight (mg) | 14.24±0.15a | 10.97±0.15b | | 14.66±0.17a | | 11.34±0.14bc | | 16.36±0.2de | | 12.11±0.13cf | | 16.79±0.24e | | 12.53±0.14f | | 20.33±0.28g | | 15.66±0.2d | | 20.66±0.16g | | 15.89±0.15de |
| Growth rate(In mg/d) | 0.26±0.002a | 0.24±0.002b | | 0.27±0.002a | | 0.24±0.002b | | 0.29±0.002c | | 0.26±0.002a | | 0.3±0.003c | | 0.27±0.002a | | 0.32±0.003d | | 0.29±0.002c | | 0.32±0.003d | | 0.3±0.002c |
|  |  |  | |  | |  | |  | |  | |  | |  | |  | |  | |  | |  |
| 28 °C | N=62 | N=81 | | N=40 | | N=44 | | N=53 | | N=55 | | N=40 | | N=45 | | N=95 | | N=101 | | N=76 | | N=80 |
| Pupal weight (mg) | 14.08±0.15ab | 10.77±0.16c | | 14.15±0.12ab | | 10.91±0.14c | | 16.16±0.2d | | 11.98±0.13e | | 16.6±0.25d | | 12.26±0.16e | | 17.95±0.24f | | 13.71±0.24a | | 18.93±0.25g | | 14.76±0.22b |
| Growth rate(In mg/d) | 0.31±0.003ab | 0.27±0.003c | | 0.31±0.003a | | 0.28±0.002c | | 0.33±0.003d | | 0.3±0.002b | | 0.34±0.003d | | 0.3±0.002ab | | 0.4±0.002e | | 0.36±0.003f | | 0.41±0.003e | | 0.37±0.003f |

Continued

| Temperature and trait | HB | |  | | SY | |  | | TA | |  | | XY | |  | | XS | |  | | LN | |
| --- | --- | --- | --- | --- | --- | --- | --- | --- | --- | --- | --- | --- | --- | --- | --- | --- | --- | --- | --- | --- | --- | --- |
|  | Female | Male | | Female | | Male | | Female | | Male | | Female | | Male | | Female | | Male | | Female | | Male |
| 16 °C | N=42 | N=47 | | N=55 | | N=57 | | N=40 | | N=42 | | N=47 | | N=51 | | N=109 | | N=108 | | N=71 | | N=73 |
| Adult weight (mg) | 12.39±0.14ab | 9.1±0.12c | | 13.19±0.17a | | 9.84±0.1c | | 16.24±0.2d | | 11.46±0.15e | | 16.64±0.2d | | 12.12±0.14be | | 19.73±0.21f | | 14.65±0.19g | | 20.38±0.21f | | 14.81±0.19g |
| Proportion weight loss | 5.72±0.41a | 8.25±0.59bc | | 6.18±0.53ab | | 10.2±0.59cd | | 6.25±0.49ab | | 10.72±0.57d | | 4.76±0.35a | | 8.9±0.53cd | | 4.22±0.53a | | 8.24±0.49bc | | 5.49±0.4a | | 9.17±0.46cd |
|  |  |  | |  | |  | |  | |  | |  | |  | |  | |  | |  | |  |
| 19 °C | N=79 | N=85 | | N=74 | | N=75 | | N=71 | | N=79 | | N=76 | | N=80 | | N=87 | | N=88 | | N=75 | | N=78 |
| Adult weight (mg) | 13.4±0.16a | 10.22±0.1b | | 13.59±0.16a | | 10.51±0.1b | | 16.58±0.2c | | 12.17±0.16d | | 16.89±0.2c | | 12.26±0.16d | | 20.93±0.22e | | 15.36±0.22f | | 21.03±0.18e | | 15.44±0.2f |
| Proportion weight loss | 7.21±0.51ab | 10.21±0.57c | | 6.92±0.33ad | | 10.36±0.43c | | 6.27±0.49ade | | 9.65±0.63c | | 5.28±0.45de | | 9.96±0.45c | | 4.6±0.34e | | 7.25±0.47ab | | 4.9±0.31e | | 8.85±0.45bc |
|  |  |  | |  | |  | |  | |  | |  | |  | |  | |  | |  | |  |
| 22 °C | N=50 | N=46 | | N=51 | | N=50 | | N=56 | | N=53 | | N=45 | | N=52 | | N=93 | | N=96 | | N=56 | | N=59 |
| Adult weight (mg) | 12.83±0.17a | 9.77±0.11b | | 13.42±0.17ac | | 10.18±0.11bd | | 15.86±0.22e | | 10.93±0.15d | | 15.94±0.17e | | 11.76±0.14f | | 19.01±0.2g | | 13.56±0.15c | | 19.46±0.15g | | 14.05±0.12c |
| Proportion weight loss | 10.5±0.39ac | 12.89±0.61a | | 7.53±0.4bd | | 12.51±0.81a | | 8.09±0.67bcd | | 13.22±0.7a | | 7.91±0.49bcd | | 10.61±0.63ac | | 7.46±0.42bd | | 10.44±0.64ac | | 6.63±0.33b | | 9.62±0.6cd |
|  |  |  | |  | |  | |  | |  | |  | |  | |  | |  | |  | |  |
| 24 °C | N=58 | N=62 | | N=60 | | N=61 | | N=60 | | N=56 | | N=51 | | N=53 | | N=67 | | N=71 | | N=54 | | N=56 |
| Adult weight (mg) | 12.78±0.13a | 9.38±0.13b | | 12.9±0.15a | | 9.74±0.13bc | | 15.06±0.2d | | 10.51±0.11ce | | 15.23±0.17d | | 10.92±0.12e | | 17.95±0.23f | | 12.82±0.17a | | 18.35±0.24f | | 13.49±0.15a |
| Proportion weight loss | 11.61±0.69ab | 14.55±0.64acd | | 10.64±0.72b | | 14.05±0.58acd | | 11.45±0.72ab | | 14.47±0.67acd | | 11.44±0.62ab | | 15.64±0.75cde | | 12.55±0.72abc | | 18.58±0.54e | | 11.42±0.63ab | | 15.89±0.57de |
|  |  |  | |  | |  | |  | |  | |  | |  | |  | |  | |  | |  |
| 26 °C | N=40 | N=46 | | N=40 | | N=40 | | N=63 | | N=62 | | N=58 | | N=55 | | N=97 | | N=103 | | N=52 | | N=58 |
| Adult weight (mg) | 12.04±0.13a | 8.97±0.12b | | 12.79±0.17ac | | 9.44±0.15bd | | 14.39±0.2e | | 10.27±0.11df | | 14.81±0.24e | | 10.41±0.15f | | 17.53±0.22g | | 12.77±0.16ac | | 18.23±0.17g | | 13.46±0.14c |
| Proportion weight loss | 14.41±0.64abd | 18.05±0.74c | | 12.7±0.68ab | | 16.68±0.9cd | | 12.06±0.6ab | | 15.18±0.52abcd | | 11.75±0.73a | | 16.9±0.85cd | | 13.48±0.79abd | | 18.25±0.73c | | 11.75±0.47a | | 15.26±0.64bcd |
|  |  |  | |  | |  | |  | |  | |  | |  | |  | |  | |  | |  |
| 28 °C | N=62 | N=81 | | N=40 | | N=44 | | N=53 | | N=55 | | N=40 | | N=45 | | N=95 | | N=101 | | N=76 | | N=80 |
| Adult weight (mg) | 12.04±0.12ab | 8.92±0.15c | | 12.44±0.12a | | 9.19±0.14cd | | 13.57±0.15e | | 9.73±0.12df | | 14.02±0.19eg | | 10.15±0.13f | | 14.58±0.2gh | | 10.52±0.21fi | | 15.08±0.19h | | 11.3±0.17bi |
| Proportion weight loss | 15.18±0.68ab | 17.13±0.7bd | | 12.07±0.45a | | 15.89±0.47ab | | 15.75±0.73ab | | 18.64±0.81bd | | 15.17±0.79ab | | 16.96±0.75bd | | 18.47±0.87bd | | 23.15±0.87c | | 19.8±1.09cd | | 23.13±0.94c |
